# Movement behaviours of stocked and wild lake trout *Salvelinus namaycush* determined using acoustic telemetry

**DOI:** 10.1111/jfb.70071

**Published:** 2025-05-02

**Authors:** Matthew H. Futia, J. Ellen Marsden

**Affiliations:** ^1^ Rubenstein Ecosystem Science Laboratory University of Vermont Burlington Vermont USA

**Keywords:** acoustic telemetry, Lake Champlain, lake trout, movement behaviour, stocking

## Abstract

Hatchery practices are a critical tool of fisheries management to supplement diminished fish populations and restore extirpated species. However, stocking programs that successfully restore self‐sustaining populations are rare. Unintentional artificial selection and domestication of hatchery‐reared fish are potential limitations to the success of stocked individuals by selecting for behaviours that are poorly suited for natural conditions. Here, we compared seasonal movement behaviours between an established population of hatchery origin (stocked) and naturally produced (wild) lake trout (*Salvelinus namaycush*) in Lake Champlain at a range of sizes, including immature and mature fish. Fifty‐six stocked and 34 wild lake trout were implanted with acoustic transmitters, including 45 transmitters with temperature and pressure (depth) sensors, to evaluate three‐dimensional movement. Movement behaviours were assessed based on the number of distinct lake regions used, proportional time spent among lake regions, average daily distance travelled and depth distribution. Overall, horizontal and vertical movements were similar between stocked and wild lake trout across sizes, although individuals tended to occupy shallower depths at larger sizes. Seasonal differences in movement behaviours were observed and in some cases were dependent on origin. For all lake trout, average daily movement was greatest during fall and least during summer. Depth occupied, however, had an opposite trend, with the deepest average depths during summer and shallowest during fall and winter. The proportion of time spent among lake regions and variability in depth occupied also varied seasonally but only for wild fish, and included less time spent in individual regions and greater depth variability during fall compared to other seasons. While origin had insignificant effects in most models we evaluated, model predictions consistently suggested stocked lake trout had slightly smaller movements than wild fish. These results suggest that hatchery practices may have long‐term, unintended effects on fish behaviour yet overall differences are likely subtle.

## 1 INTRODUCTION

Fish stocking has long been used as a management tool for augmenting fisheries and more recently has become a common practice for fish conservation by supplementing native populations. These types of stocking programs originate from different goals, supporting fish populations for harvest versus recovery, but have converged on their objective to produce quality fish with sufficient fitness to survive in natural settings (Claussen & Philipp, 2023; Trushenski et al., 2010). Programs that support native populations are typically established to promote recovery of self‐sustaining wild populations; however, this goal is frequently not met and stocking practices have been associated with negative impacts on remnant wild populations (Araki & Schmid, 2010; Claussen & Philipp, 2023; Hagen et al., 2021). Numerous factors have been identified that can inhibit restoration of self‐sustaining populations (e.g. prey availability, habitat quality, genetic diversity, etc.; Cochran‐Biederman et al., 2015; Uusi‐Heikkilä et al., 2018), and unintended domestication and altered fish behaviours associated with hatchery rearing have frequently been identified as critical limitations of stocking programs (e.g. Araki et al., 2008; Araki & Schmid, 2010; Chittenden et al., 2010; Thériault et al., 2011).

Early‐life‐stage experiences influence animal development as individuals actively and passively respond to ambient conditions with phenotypic plasticity. Additional responses can occur epigenetically, causing rearing environment to further influence fitness‐related traits (Le Luyer et al., 2017). This sensitivity to environmental conditions often includes behavioural responses and can result in irreversible, life‐long consequences (DePasquale et al., 2016; Nyman et al., 2020; Taborsky, 2016; West‐Eberhard, 2005). Hatchery‐reared fish often behave differently than wild conspecifics, including altered habitat selection and distribution (e.g. Bolland et al., 2008; Jonsson et al., 1991;Kallio‐Nyberg et al., 2011; Tatara et al., 2009). Reduced selection in hatcheries resulting from high survival relative to natural environments can also lead to altered behaviours in hatchery fish compared to wild individuals (Araki et al., 2008). The extent of these differences, however, can be species‐ and context‐dependent, with stocked fish shown to have both wider (Bolland et al., 2008; Jonsson et al., 1991) or reduced distributions (Kallio‐Nyberg et al., 2011; Tatara et al., 2009) compared to wild conspecifics. Most studies to date have evaluated behavioural differences at specific life stages, especially in the context of fish distributions.

Here, we investigated the lifetime influence of hatchery rearing on fish movement by evaluating the distribution of stocked and wild lake trout *Salvelinus namaycush* (Walbaum 1792) across life stages in a large lake system (Lake Champlain, USA). Lake trout are a mobile, cold‐water species that typically occupy deeper habitats where cool water is available (Marsden et al., 2021). Individuals can disperse widely throughout systems, often in response to prey availability, reproduction and habitat conditions (Binder et al., 2021). Lake trout can grow to large sizes (>1 m) and typically mature around 8 years based on age (in years) at 50% maturity across multiple systems (Hansen et al., 2021). In Lake Champlain, lake trout were extirpated from the lake by the 1900s through undetermined causes, but stocking programs in the 1970s re‐established a population that was comprised entirely of stocked fish for the following four decades (Marsden & Langdon, 2012). Juvenile lake trout (ages 0 and 1 year) were stocked in the lake annually with gametes largely originating from a feral population in the Finger Lakes of New York, which is comprised primarily of stocked fish, and from locally‐sourced captive broodstock made up of offspring from lake trout stocked in Lake Champlain (Ellrott & Marsden, 2004). Stocking efforts created an abundant fishery with stocked lake trout surviving to 30+ years (Hemmelgarn et al., 2022; Marsden & Langdon, 2012). Stocked fish that matured successfully spawned at multiple locations in the lake leading to substantial production of free embryos (Ellrott & Marsden, 2004; Marsden et al., 2005). However, the first evidence of substantial natural recruitment was not observed until the 2010s (Marsden et al., 2018). By the early 2020s, wild fish were common in the lake, with multiple cohorts of adults up to 10 years old, providing an opportunity to compare behaviours between stocked and wild fish at various years post‐stocking. Recent studies have also identified behavioural differences between stocked and wild juveniles in the lake, including habitat use (Wilkins & Marsden, 2021), prey selectivity and foraging success (Marsden et al., 2022).

The objectives of this study were to evaluate differences in the spatial extent of stocked and wild lake trout movements, including depth distribution, and determine whether such differences are retained for extended periods (i.e. years) after hatchery‐reared fish are released. We also examined seasonal patterns in lake trout distribution and habitat use that have been observed in other systems (Blanchfield et al., 2023; Funnell et al., 2023; Gallagher et al., 2019; Ivanova et al., 2021). We addressed these objectives using acoustic telemetry to track horizontal and vertical movements of stocked and wild lake trout at a range of sizes, from immature juveniles to mature adults, throughout Lake Champlain across multiple years.

## 2 MATERIALS AND METHODS

### 2.1 Study system

**FIGURE 1:**
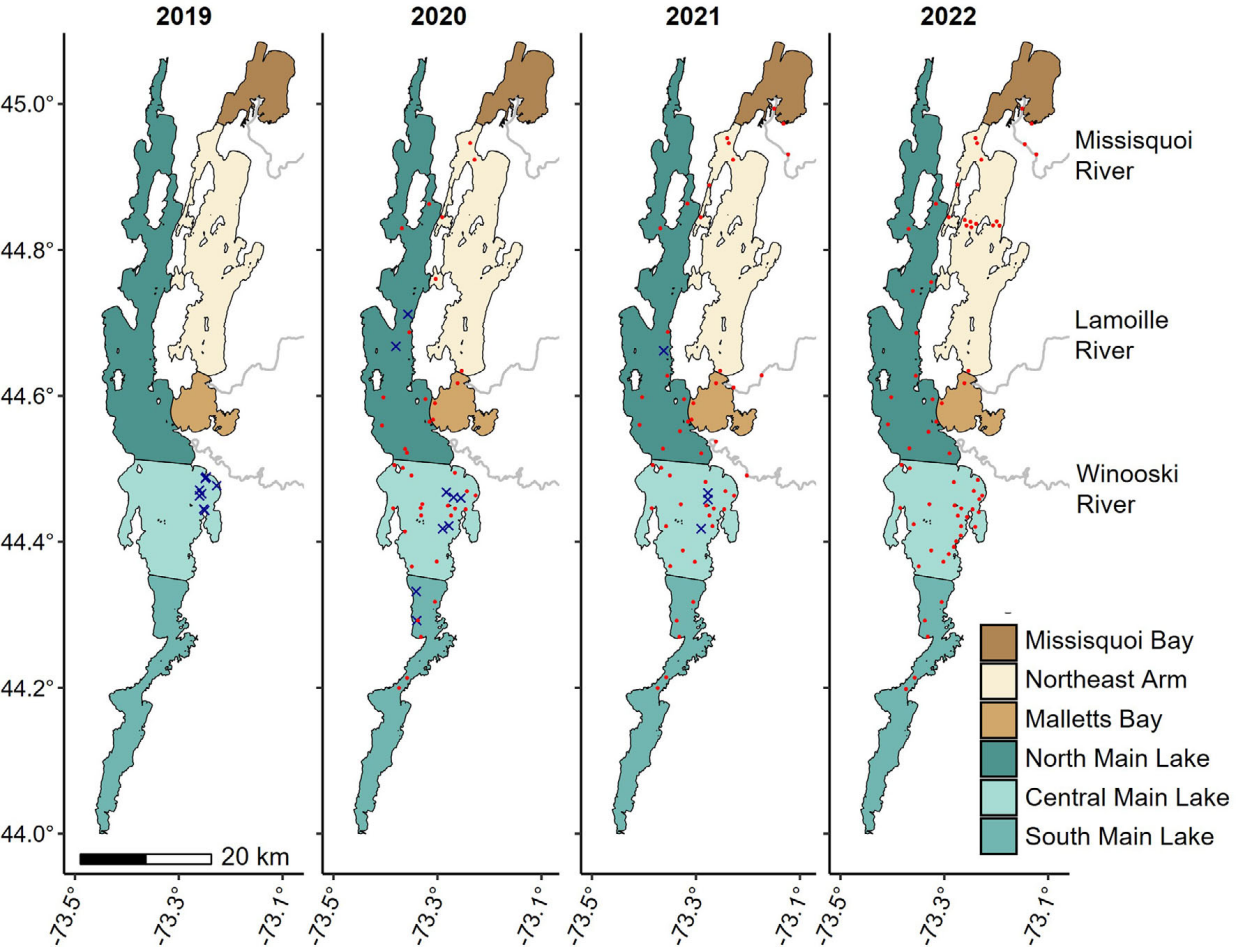
Maps of Lake Champlain, coloured by lake region, with receiver station locations (red points) and lake trout (*Salvelinus namaycush*) sampling locations (blue crosses) included in their corresponding deployment and capture years, respectively. Lake tributaries with receiver deployments are also included. Each receiver location point has a radius of 500 m, corresponding to approximately 30% detection efficiency from Pinheiro et al. (2017). The South Lake region is excluded from the maps because no receivers were deployed nor fish captured throughout this region.

Lake Champlain is situated between New York and Vermont, USA with the northern‐most sections extending into Quebec, CAN. The lake has five distinct basins largely separated by islands and human structures (i.e. causeways; Figure 1). The largest basin, referred to as the Main Lake, is primarily oligotrophic and has three separate management zones that vary in maximum depth. The southern region of the Main Lake (South Main Lake) includes the deepest habitats in the lake (approximately 122 m), the central region (Central Main Lake) has a maximum depth of 106 m, and the northern region North Main Lake has a maximum depth of 101 m. Missisquoi Bay and the South Lake are mostly shallow (<15 m) and eutrophic, whereas Malletts Bay (maximum depth = 34 m) and the Northeast Arm (maximum depth = 49 m) are mesotrophic and often experience hypoxia in offshore waters during summer stratification (Marsden & Langdon, 2012; Smeltzer et al., 2012).

### 2.2 Fish collections

Lake trout were collected between October 2019 and September 2021, and included 56 stocked and 34 wild (i.e. naturally produced) individuals (Table 1). Stocked fish were distinguished from wild individuals by the absence of a fin (i.e. clipped paired fin or adipose fin used to identify hatchery fish) with a clipping error rate of approximately 2% (Marsden et al., 2018). All stocked lake trout that were tagged had been living in the lake for more than 1 year prior to tagging based on age‐at‐length estimates at the time of tagging and considering lake trout are only stocked as fall fingerlings and spring yearlings. Fish shorter than 350 mm were collected from the three Main Lake regions using bottom trawls (10 min tow using a 3‐in‐1 trawl). All fish longer than 350 mm, both immature and mature, were captured from the central Main Lake region primarily by angling (jigging and trolling) in addition to gillnets (overnight set using 63.5–88.9 mm stretch mesh) and bottom trawling (Table 1 and Figure 1). All fish were captured between July and November, with most caught during summer months, especially fish longer than 350 mm (Table S1). After capture, lake trout considered unhealthy or with abnormal conditions (e.g. sea lamprey *Petromyzon marinus* wounding or barotrauma) were released while fish to be tagged were held in chilled (~10°C) lake water. Total length was measured for each fish and was used to determine maturity status using a cut‐off for maturity at 500 mm (i.e. immature fish were ≤ 500 mm) based on size‐at‐maturity data for 1343 lake trout collected from Lake Champlain in gillnets deployed by the Vermont Fish and Wildlife Department (VTFWD) in summer 2020–2023. The cut‐off was assigned as the smallest 25 mm size bin with >50% maturity for stocked and wild males and females (Figure S1). Although size at maturity differed between males and females based on the VTFWD dataset, we were not able to determine sex for individuals tagged in this study and therefore used a single size threshold.

**TABLE 1:** Collection data for lake trout (*Salvelinus namaycush*) tagged with acoustic transmitters by origin, life stage, collection method, transmitter type, capture region (North Main Lake = NML, Central Main Lake = CML, South Main Lake = SML) and months collected.

Origin	Life stage	Method	Transmitters	Regions	Months
Stocked	Juvenile	Trawl	V9TP (11)	NML, CML, SML	August, October
	Sub‐adult	Angling	V9TP (2), V13 (8)	CML	August‐November
	Adult	Angling	V9TP (15), V13 (16)	CML	July‐November
		Gill net	V13 (4)	CML	October
Wild	Juvenile	Trawl	V9TP (5)	CML	October
	Sub‐adult	Angling	V9TP (5), V13 (14)	CML	July‐November
		Gill net	V13 (1)	CML	October
	Adult	Angling	V9TP (5), V13 (1)	CML	September, November
		Trawl	V9TP (2), V13 (1)	CML	August, November

*Note*: Sample sizes are included in parentheses for transmitter type by origin, life stage, and capture method; complete samples sizes also accounting for region and collection month are included in Table S1.

### 2.3 Acoustic telemetry tracking

The acoustic receiver array in Lake Champlain consisted of 37 VR2W and two VR2Tx receivers (69 kHz; Innovasea) that were deployed in the Main Lake, Malletts Bay, and Northeast Arm during summer 2020. An additional 10 VR2W receivers were added to the array during 2021 including receivers in the Missisquoi (*n* = 2), Lamoille (*n* = 2) and Winooski rivers (n = 2), Missisquoi Bay (*n* = 1), the Northeast Arm (n = 1) and the North Main Lake (*n* = 2). Eleven more receivers, including one VR2W and 10 VR2Tx receivers, were deployed during 2022 primarily in the Central Main Lake region. In addition to positioning receivers in the broad lake, receivers were deployed strategically at narrow passages between lake regions, previously identified lake trout spawning locations (Ellrott & Marsden, 2004) and near abrupt underwater rises (lakemounts) that may serve as important ecological hotspots (Possamai et al., 2024). Multiple concurrent studies used the receiver array, causing some receivers to be repositioned during the study, including intermittent coverage in tributaries where receivers were only deployed during winter and spring (Figures 1 and S2). Receivers were either suspended from subsurface buoys attached to mooring anchors for deep deployments or directly attached to mooring anchors in tributaries and shallow locations. Both receiver models use the same technology to detect transmissions and therefore were expected to have comparable performance for detecting transmissions under similar environmental conditions. The VR2Tx receivers have additional sensors and collected temperature data at 1 h. intervals and can send transmissions, although this feature was deactivated for this study to preserve receiver battery life.

Two types of acoustic transmitters were used in the study: 45 V9TP transmitters (69 kHz, 146 dB re 1 μPa at 1 m, 525‐ or 715‐day battery life) with temperature and pressure (used to calculate depth) sensors and 45 V13 transmitters (69 kHz, 147 dB re 1 μPa at 1 m, 1354‐day battery life) with no environmental sensors (Innovasea). All transmitters had a random transmission delay ranging between 80 and 160 s. Fish were immobilized using electroanesthesia (Reid et al., 2019) for surgical implantation of the transmitter and a laminated disk anchor for an external identification tag (FM‐89SL; Floy Tag & Manufacturing Inc.). Electroanesthesia was performed using a transcutaneous electrical nerve stimulation unit (TENS 7000) administering a pulsed direct current between an anode electrode held near the head and cathode electrode near the caudal fin. Transmitters were implanted in the body cavity though a small ventral incision parallel and next to the linea alba. Once the transmitter and disk anchor were in place, the incision was closed with two or three simple interrupted sutures using coated Vicryl braided suture material (Eithicon Inc.; Wagner et al., 2011). Fish were partially submerged in lake water chilled to ~10°C during surgery so that water flowed over the gills while keeping the surgery site dry. Lake trout recovery from electrosedation is rapid (~200–400 s; Faust et al., 2017) and fish were held in the chilled lake water for 2–5 min prior to release to assess post‐surgery recovery and potential tag loss. Overall, 56 stocked lake trout were tagged with 28 V9TP and 28 V13 transmitters and 34 wild fish were tagged with 17 V9TP and 17 V13 transmitters (Table 1). Transmitters used with lake trout in this study had an estimated 70% DE (detection efficiency; Brownscombe et al., 2020) of 250 m based on range testing conducted for previous studies in Lake Champlain that used transmitters with similar program options (Pinheiro et al., 2017; Supplementary Text). However, this DE was not incorporated into statistical analyses as our study focused on broad‐scale regional movements rather than distribution at individual receiver locations.

Data from all acoustic receivers were offloaded annually. Detection data were time‐corrected using VUE software (Innovasea) to account for clock drift. Data were then filtered to remove potentially erroneous detections characterized as transmitters with a single detection at a given receiver throughout a period longer than 1 h (2% of detections) (Simpfendorfer et al., 2015). Data were also filtered to remove (1) detections during the season in which fish were released to account for any differences in behaviour associated with transmitter implantation (Rogers & White, 2007), with no fish tagged less than 7 days before the end of a season, (2) fish considered to be dead, which were characterized by highly repeated observations at a single receiver until either the end of the study or transmitter battery lifespan, or individuals with V9TP transmitters that showed no vertical activity (i.e. standard deviation of <1 m for all depth data), (3) detections prior to summer 2020 due to the substantial shift in the receiver array, (4) detections after winter 2023 when the final receiver offloads occurred and (5) fish that were not detected for at least one full season following the previous filtering steps. A total of 3,485,162 detections among 75 individuals remained after data filtering.

### 2.4 Animal ethics

The protocols used for fish handling and surgery in this study were approved by the Institutional Animal Care and Use Committee at the University of Vermont (protocol # 20–004) following guidelines of the US Public Health Service Policy and the Animal Welfare Act.

### 2.5 Life stage and season determination

Total length of tagged lake trout and seasonal thermal conditions of the lake (i.e. extent of stratification) were both estimated at the time of all detections. Daily growth of tagged lake trout was accounted for by estimating daily increases in length relative to the length at capture using growth rates determined for stocked and wild lake trout in Lake Champlain (Hemmelgarn et al., 2022). Five stocked lake trout were larger than the estimated theoretical maximum length (*L*∞) from Hemmelgarn et al. (2022) when they were captured, and their estimated lengths were held constant throughout their detection period. Maturity status for each lake trout was determined daily from length estimates to identify when individuals transitioned to maturity for statistical analyses. Median predicted growth throughout the study period was 62 mm and maximum predicted growth among all fish was 139 mm.

Dates associated with seasons that were biologically relevant to lake trout (i.e. represented changes in experienced environmental conditions; Ivanova et al., 2021) were determined annually using data collected from (1) V9TP transmitters representing temperatures experienced by lake trout and (2) a US Geological Survey station in Burlington, VT in the Central Main Lake that recorded lake temperature approximately 3 m below the surface at 15‐min intervals (US Geological Survey, 2024). The coldest daily temperatures experienced by lake trout were determined for each fish and averaged to generate a daily experienced temperature. Minimum temperatures were used rather than averages to better identify the coldest temperatures available. Near‐surface temperatures were summarized as average daily temperature. Temperatures experienced by fish and near‐surface temperatures were compared daily to identify periods when the relationship between these two temperatures differed, with patterns in this relationship driven by changes in thermal stratification (presence/absence and depth of the thermocline) and lake trout selection for cold habitats located below the thermocline. These periods were used to define biological seasons that were used in analyses. Winter was defined as cold periods when near‐surface temperature and lake trout experienced temperature were nearly equal. The onset of spring was identified as the period when experienced temperatures deviated from near‐surface temperature indicating development of thermal stratification. Summer was defined as the period when experienced temperatures were consistent and differences from near‐surface temperatures were greatest. Lastly, the start of fall was identified by convergence of the two temperatures that indicated breakdown of the thermocline and ended when the near‐surface temperature dropped below the temperature identified to distinguish winter from spring. The lake temperatures associated with these patterns were used as thresholds for biological seasons and extrapolated across all years throughout the study, including seasons when lake trout temperature data were not collected. A 7‐day rolling average of lake temperature was used to smooth daily temperature changes and provide hard transitions between seasons.

### 2.6 Statistical analyses

All statistical analyses were conducted using R software (R Core Team, 2023). Patterns in lake‐wide distribution of lake trout were evaluated based on the number of lake regions used (region use), proportion of time spent in each lake region (regional occupancy) and horizontal distance travelled (average daily movement). Depth distribution was assessed based on the average daily depth and daily standard deviation of depth (vertical activity; Gallagher et al., 2019). For each analysis, lake trout were grouped by fish origin (stocked or wild) and the biological season at the time of detection. The lake region where detections occurred was also included for evaluations of average daily movement and depth distribution to account for regional differences in receiver density. Data used for assessing patterns in depth distribution were filtered for each individual to only retain dates that had more than 20 detections with depth data in a given region. Average depth and vertical activity were then determined daily using 20 randomly selected values for each fish and day to maintain consistent sample sizes for fish depth and avoid biasing standard deviation calculations. Randomly subsampling data can also help reduce effects of autocorrelation (Brownscombe et al., 2022).

Regional distribution was assessed using modelled fish paths generated with the *glatos* package (Holbrook et al., 2024) as described by Futia et al. (2024). Briefly, lake trout positions were generated at 1‐h timesteps based on the time of detections at receiver locations and using a combination of linear and non‐linear interpolated positions between subsequent detections to create the shortest paths between receivers that avoided land. The longest gap between subsequent detections for an individual fish was 789 days (the longest gap for all other fish was less than 365 days), but the average gap with one standard deviation was only 22 min ± 18 h. While it is possible for fish to move between regions during extended gaps, this was unlikely given the coverage of acoustic receivers, especially between regions. We therefore expect that undetected fish remained in smaller areas that lacked receivers within individual regions and retained all estimated positions. These interpolation data were used to calculate (1) region use as the number of unique regions in which an individual was detected during each season and (2) regional occupancy as the proportion of time present in each region (Futia et al., 2024) also during each season. The most used region (i.e. maximum regional occupancy) for each individual and season was selected for comparisons and was used to represent the extent of cross‐region movement (values closer to one indicate high dependence on a single region). Significant covariates of region use and regional occupancy were evaluated using generalized linear mixed effects models (GLMMs) with the R package ‘glmm_TMB’ (Brooks et al., 2017). Origin, transmitter type (V9TP or V13), biological season, an interaction term between season and origin, and estimated total length at the day of detection averaged by season (estimated length) were included as fixed effects in the global model for region use. Maximum regional occupancy was also evaluated based on these covariates except estimated length was replaced with estimated maturity status, which allowed better model convergence and fit. Both global GLMMs also included an identifier variable for individual fish, detection year and number of days with an estimated position for individual fish during a given season as random effects. The number of days with estimated positions within a given season was included as a random effect to account for variation in duration among biological seasons and for individuals that did not have position estimates throughout a season. The GLMM used for evaluating region use followed a Poisson distribution considering the values represented count data and data were not overdispersed. Regional occupancy was calculated as a continuous probability and evaluated using a GLMM with a beta distribution. Estimates of regional occupancy included values of 1 and therefore required transformation (slight compression of range of values by approximately 0.001) to fit the beta distribution (Smithson & Verkuilen, 2006).

Average daily movement was estimated based on movement among approximate positions based on centres of activity (COA) generated at 1‐h intervals (Simpfendorfer et al., 2002). The COAs were assigned to 1‐km polygons, which were constructed as a hexagonal grid overlaying the lake (Birch et al., 2007), and movement between polygons was calculated as the distance between polygon centroids. Most polygons (97%) did not include any receivers and of the polygons that did encompass receivers, one polygon included two receiver stations, and the rest only contained a single station (Figure S3). This grouping of adjacent COAs within 1 km polygons was conducted to remove the effects of small, local movements and allowed us to focus on larger movements that we assumed represented movement to new areas. Seasonal movement for each lake trout was calculated as the total distance travelled among polygons within each season. Average daily movement was then calculated for each fish as the seasonal distance travelled divided by the number of days between approximate positions during the season. Average daily movement was evaluated using GLMM analysis following a Tweedie distribution to account for zero inflation of the response variable (Dunn & Smyth, 2005). Origin, estimated length, transmitter type and biological season were again included as fixed effects and lake trout identification numbers, detection year and lake region were included as random effects in the global model. Lake region was included as a random effect to incorporate a spatial component into the model that may be associated with inconsistent receiver coverage across regions.

Factors associated with average daily depth and vertical activity were also evaluated using GLMMs. The global models incorporated origin, biological season, interactions between origin and season, and estimated length at the day of detection as fixed effects. Both global GLMMs also included lake trout identification number, number of days with more than 20 depth recordings for individual fish during a given season and lake depth as random effects. Lake depth was calculated as the average depth of receiver stations involved with each subset of 20 detections and was included to account for spatial differences in vertical habitat availability. The GLMM evaluating average depth was generated with a lognormal distribution and the GLMM evaluating vertical activity was constructed using a Tweedie distribution to account zeros in the data.

Optimal GLMMs were selected from each global model by comparing estimates among GLMMs using all combinations of fixed effects with the *MuMIn* package in R (Bartoń, 2023). The optimal model was chosen based on parsimony and model fit in which the GLMM that incorporated the fewest parameters (most parsimonious) while maintaining an Akaike Information Criterion (AIC) score within two units of the best fit model was selected. Significant fixed effects were identified based on an alpha of 0.05. Pairwise comparisons of significant parameters and interactions were investigated based on model predictions using estimated marginal means from the *emmeans* package in R (Lenth, 2023) and significance was tested using Tukey adjustments. Estimated effects of significant parameters were predicted based on the optimal model using the predict function from the *stats* package in R (R Core Team, 2023). Given our hypotheses were focused on comparisons between stocked and wild fish, we also evaluated models that included origin and had an AIC score within two units of the optimal model to assess trends based on lake trout origin.

## 3 RESULTS

### 3.1 Biological seasons

**FIGURE 2:**
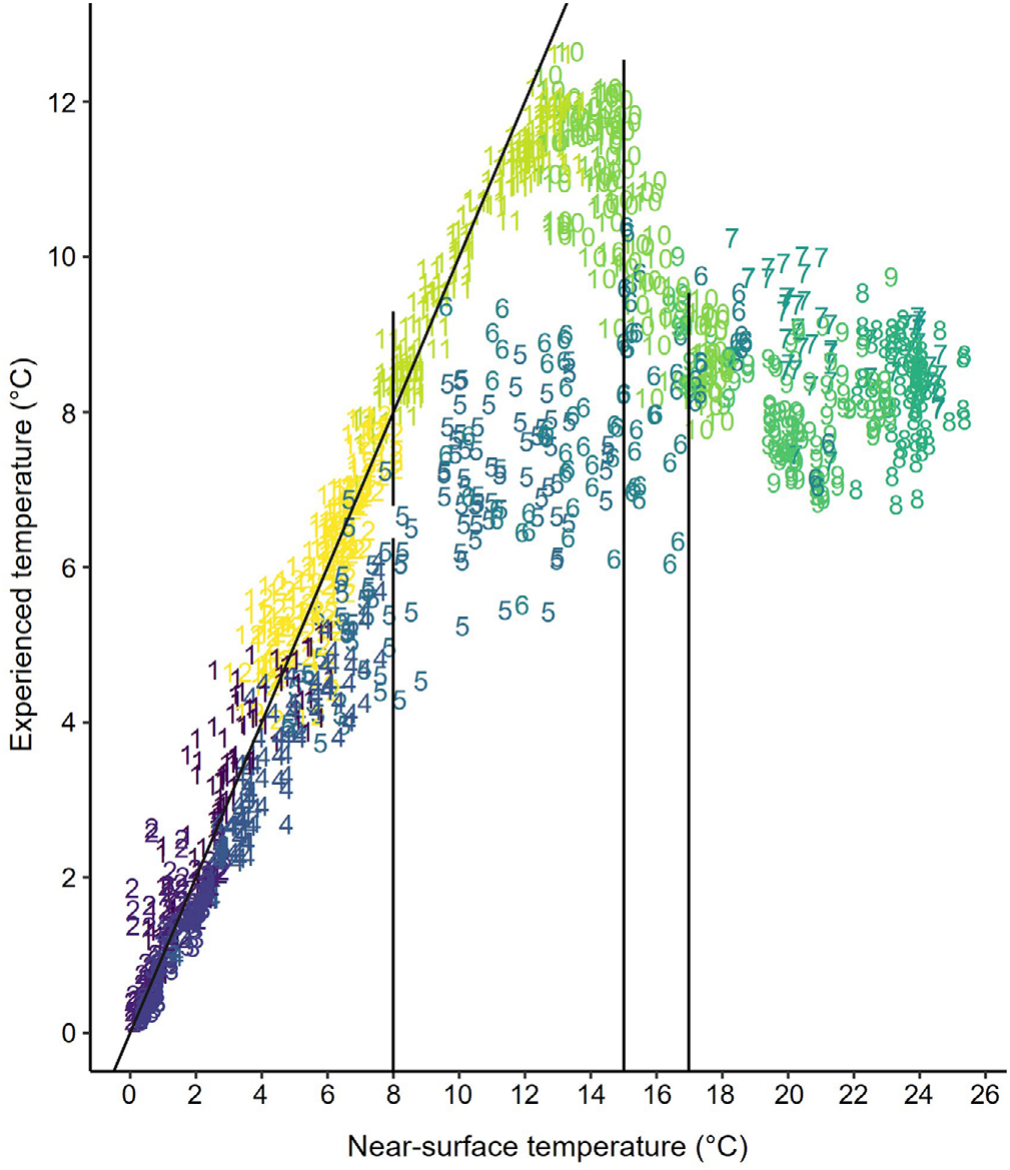
Correlations between daily lake temperature and minimum temperature experienced by lake trout (*Salvelinus namaycush*) in Lake Champlain between July 2021 and August 2023. The colour and number of each point correspond to month number. Each point represents the average minimum temperature among all lake trout (including immature and mature fish) with active V9TP transmitters (*n* = 5–29) for a given date. Vertical bars represent near‐surface temperature cutoffs used to distinguish seasons.

Average daily minimum temperature experienced by lake trout was typically consistent between maturity stages and by origin with the maximum daily differences in temperature among groups (i.e. stocked versus wild and immature versus mature) less than 1°C (median maximum difference = 0.60°C), therefore lake trout temperatures were not grouped by life stage or origin when compared to ambient, near‐surface lake temperature. Temperatures experienced by lake trout were lowest during January and February and highest during October and November. During December through March, experienced temperatures were closely aligned with the near‐surface water temperature. Experienced temperatures began to deviate from the near‐surface temperatures during late April when surface temperatures approached 8°C, demonstrating thermal stratification with lake trout occupying cooler habitats. Experienced temperatures tended to increase through June but at a slower rate than the near‐surface temperature. During late June, after near‐surface temperatures exceeded 17°C, experienced temperatures stabilized and became most distinct from the near‐surface temperatures, indicating the presence of a strong thermocline. Experienced temperatures began to rise in early October as thermal stratification broke down and near‐surface temperatures fell below approximately 15°C; experienced and near‐surface temperatures converged by the end of the month. After October, experienced and near‐surface temperatures were isothermal and dropped consistently until reaching a minimum during February (Figure 2). Based on these results, biological seasons using near‐surface water temperature data were defined as winter, below 8°C (168 ± 13 days), spring, 8–17°C (37 ± 9 days), summer, above 17°C until near‐surface temperature drops below 15°C (119 ± 14 days), and fall, 8–15°C following summer (45 ± 11 days).

### 3.2 Lake‐wide distribution

Data filtering eliminated detections from 15 individuals including 11 stocked (five immature and six mature) and four wild (all immature) lake trout. Seven of these fish were considered to have died or lost their transmitter and the remaining eight were removed due to lack of positions. The remaining lake trout included 45 stocked fish, 13 immature and 32 mature, and 30 wild fish, 15 immature and 15 mature. Of these remaining fish, six stocked and 12 wild immature lake trout transitioned to maturity during their detection period; two wild and one stocked lake trout matured before being detected as a juvenile. No lake trout were detected at any receivers deployed in lake tributaries (i.e. Missisquoi, Lamoille and Winooski rivers) nor in Missisquoi Bay during the 2022 and 2023 deployments and therefore these locations were excluded from all analyses.

**FIGURE 3:**
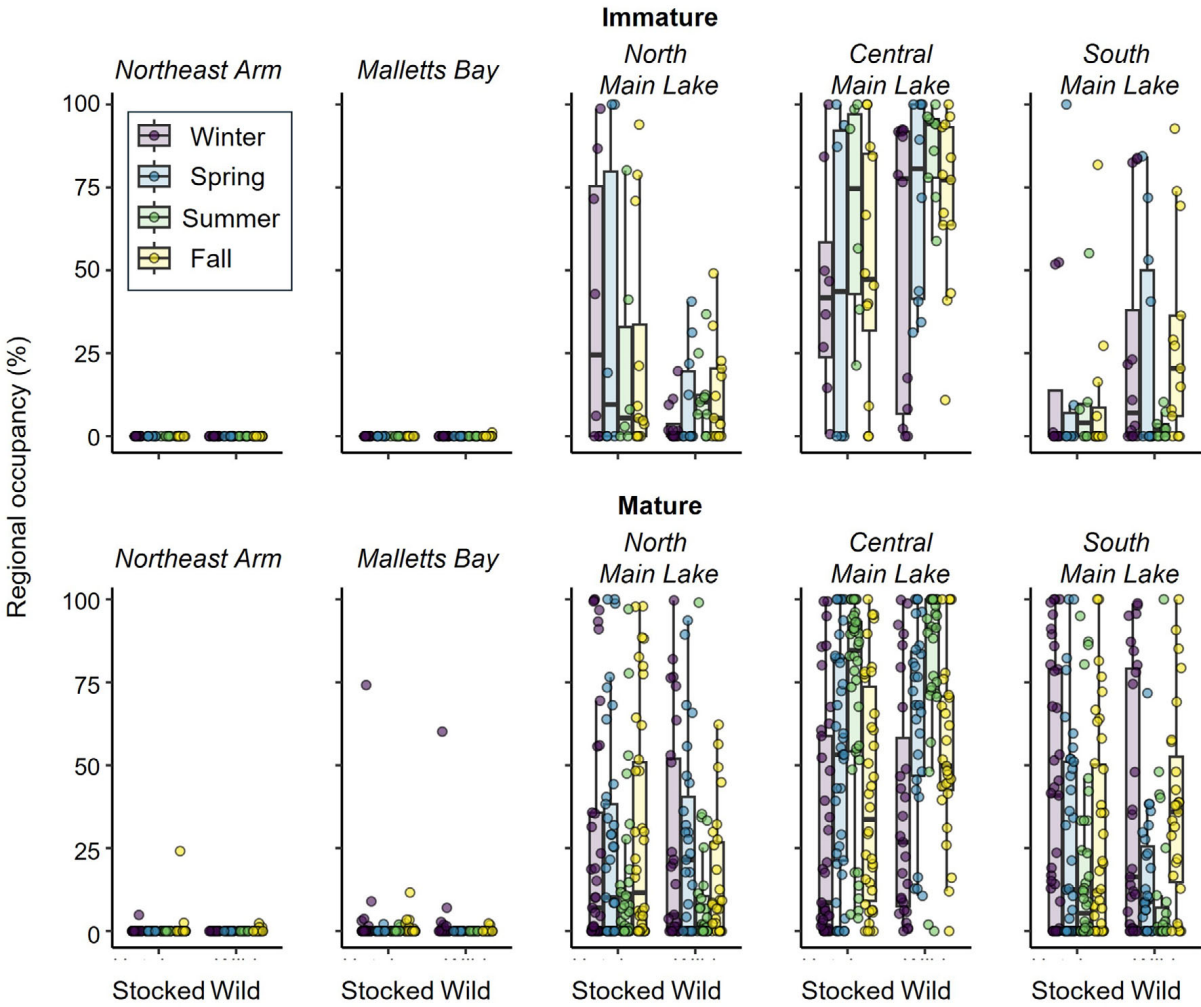
Seasonal lake trout (*Salvelinus namaycush*) distribution among five regions of Lake Champlain separated by maturity and origin (stocked or wild) and estimated from interpolated fish paths. Regional occupancy is calculated as the percentage of time individuals spent in the defined region for a given season, averaged across years when possible.

The three Main Lake regions were each used by most (≥ 66%) of the tagged fish for both maturity stages and origins. Malletts Bay was used by only 15 lake trout, including only one immature fish, and primarily during winter. Similarly, the Northeast Arm was only used by five mature lake trout and only during winter and fall (Table 2 and Figure 3). All three Main Lake regions were used by both immature and mature lake trout throughout the year. Immature fish distributions varied by origin and season with stocked fish spending a greater proportion of their time on average in the North Main Lake, especially during winter, whereas wild fish spent more time in the South Main Lake in all seasons except summer. Regardless of these differences, both stocked and wild immature fish used the Central Main Lake region the most in all seasons (Figure 3). Mature fish distributions throughout the Main Lake regions varied less by origin compared to immature fish but still varied seasonally. Most stocked and wild fish primarily used the central region of the Main Lake during spring and summer and had more homogeneous distributions throughout the Main Lake during winter and fall (Figure 3).

**TABLE 2:** Lake trout (*Salvelinus namaycush*) distribution among five regions of Lake Champlain (Northeast Arm = NEA, Malletts Bay = MAB, North Main North = NML, Central Main Lake = CML, South Main Lake = SML) estimated from interpolated fish paths and grouped by maturity (immature ≤500 mm, mature >500 mm) at the time of detection and origin.

Region	Maturity	Stocked			Wild		
		Number of fish	Number of days	Percentage of days	Number of fish	Number of days	Percentage of days
NEA	Immature	0 (12)	0.0 ± 0.0	0.0 ± 0.0	0 (13)	0.0 ± 0.0	0.0 ± 0.0
	Mature	2 (38)	0.1 ± 1.2	0.2 ± 2.0	3 (27)	0.0 ± 0.2	0.0 ± 0.4
MAB	Immature	0 (12)	0.0 ± 0.0	0.0 ± 0.0	1 (13)	0.0 ± 0.1	0.1 ± 0.4
	Mature	7 (38)	1.2 ± 10.4	0.9 ± 6.5	8 (27)	0.9 ± 7.6	0.6 ± 4.8
MLN	Immature	9 (12)	28.1 ± 46.0	33.9 ± 41.5	12 (13)	6.6 ± 10.6	8.9 ± 13.0
	Mature	32 (38)	22.2 ± 38.8	25.8 ± 35.5	26 (27)	19.7 ± 36.0	19.9 ± 27.7
MLC	Immature	11 (12)	46.3 ± 43.5	54.3 ± 39.5	13 (13)	60.4 ± 53.8	66.3 ± 34.3
	Mature	38 (38)	41.5 ± 44.9	46.9 ± 38.6	27 (27)	48.9 ± 43.4	56.7 ± 35.2
MLS	Immature	8 (12)	12.6 ± 27.5	13.5 ± 26.7	11 (13)	22.9 ± 39.7	25.7 ± 32.4
	Mature	32 (38)	27.5 ± 43.2	29.6 ± 36.0	24 (27)	25.5 ± 43.5	26.5 ± 32.9

*Note*: Distribution for each region is shown as the number of fish present (of total fish, shown in parentheses), the number of days during a season spent in each region averaged across individuals (mean ± 1 standard deviation) and the percentage of days during a season spent in each region averaged across individuals (mean ± 1 standard deviation).

**FIGURE 4:**
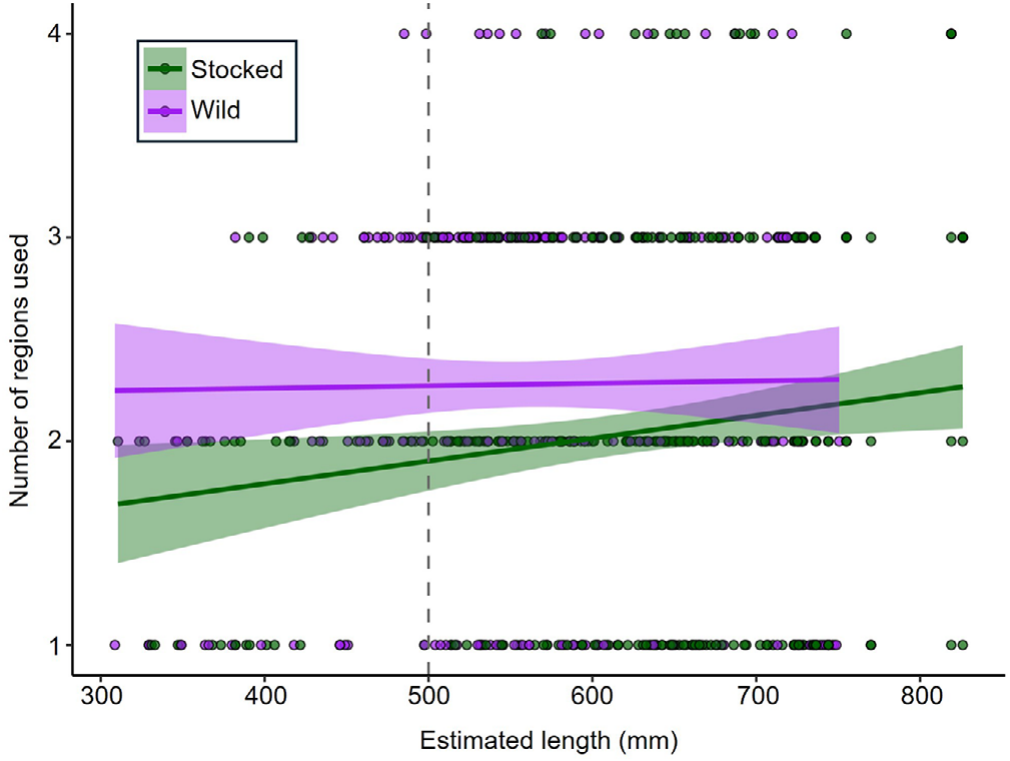
Relationship between the number of distinct regions used per season and fish total length for stocked and wild lake trout (*Salvelinus namaycush*). The linear line of best fit with 95% confidence intervals is included for each group (stocked or wild). The grey vertical dashed line at 500 mm represents the approximate size at maturity for this lake trout population.

All tagged fish were detected in at least two regions throughout their entire detection period with an overall average of 3.0 ± 0.7 (average ± one standard deviation) regions used. The optimal GLMM based on AIC score and model parsimony for evaluating seasonal region use excluded all parameters that were included in the global model. However, origin was included in the top two models based on AIC scores alone (Table S3). Still, origin did not have a significant effect (*p* = 0.091; GLMM) based on estimates from the best fitting model that included origin (Figure S4). The predicted effect of origin based on this GLMM including origin was an approximately 15% increase in number of regions used per season by wild fish (predicted region use = 0.82 regions) compared to stocked fish (predicted region use = 0.71 regions) (Figure 4).

**FIGURE 5:**
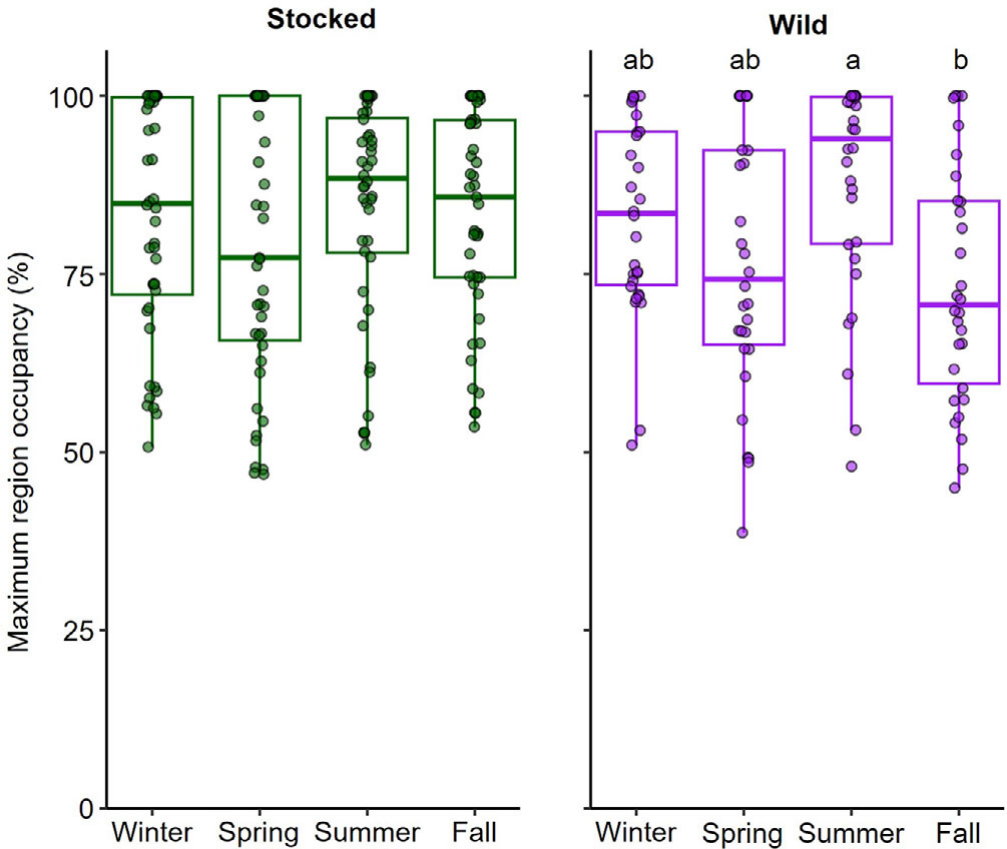
Maximum percentage of time that individual lake trout (*Salvelinus namaycush*), grouped by origin (stocked or wild), spent in their most occupied region during each biological season detected. Data were averaged across years by season for fish with multiple years of detections. Significant differences among seasons, denoted by differing letters and based on estimated marginal means, are shown for wild lake trout; significance is not shown for stocked fish as there were no differences among seasons. Boxplots include the median values (horizontal bar) and interquartile range (box) with error bars representing most extreme values within 1.5 times the interquartile range.

Most lake trout spent the majority of each season within a single region with the overall average percentage of time spent in the most‐used region during a given season equal to 81.2 ± 10.2%. The optimal GLMM evaluating maximum regional occupancy included origin, season and the interaction between these two parameters (Table S3). Both origin and season had insignificant effects individually (*p* > 0.664, GLMM; Figure S4), but maximum regional occupancy differed significantly among seasons depending on lake trout origin. Stocked fish had statistically similar maximum regional occupancy among all seasons (*p* > 0.999, Tukey). Wild fish, however, had significantly greater maximum regional occupancy during summer (predicted percentage of time = 83%) compared to occupancy during fall (predicted percentage of time = 75%, *p* = 0.004); no other pairwise comparisons between seasons were significantly different for wild fish (*p* > 0.070; Figure 5). Seasonally, there were no significant differences in any pairwise comparisons between stocked and wild fish for each season (*p* > 0.101, Tukey).

**FIGURE 6:**
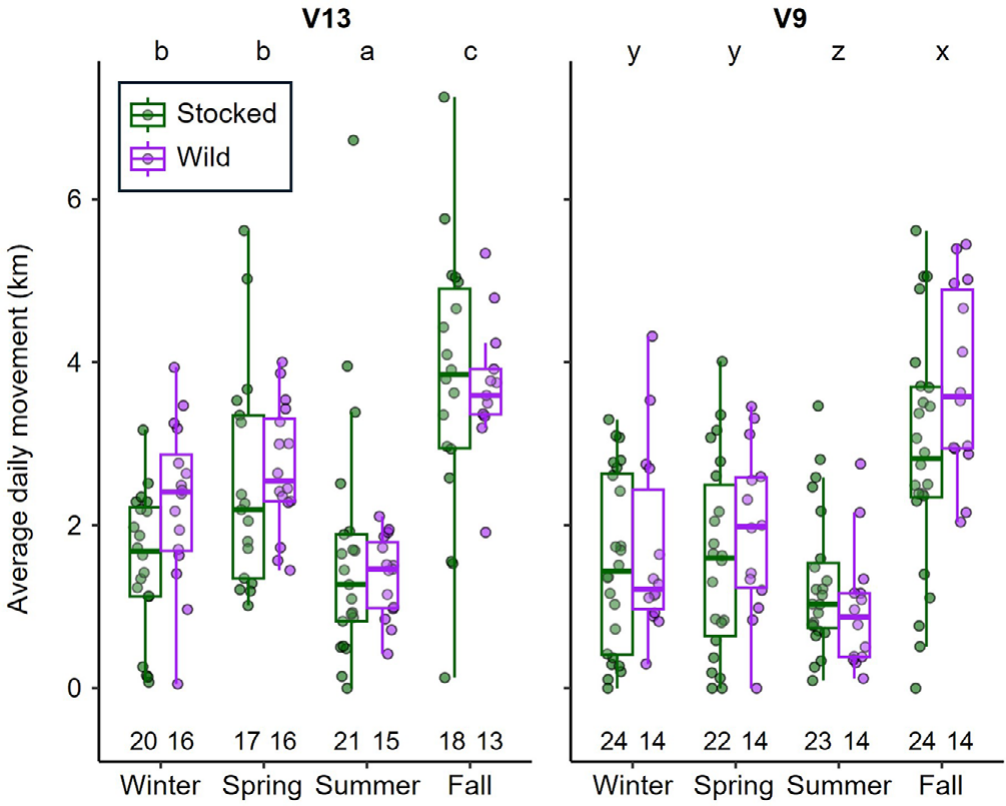
Average distance travelled daily by lake trout (*Salvelinus namaycush*) among 1‐km polygons during each biological season detected. Lake trout are grouped by origin (stocked or wild) and type of acoustic transmitter used, and data were averaged across years by season for individuals with multiple years of detections. Significant differences among seasons within each tag type, based on estimated marginal means, are denoted by differing letters. The number of fish within each group is included below each box. Boxplots include the median values (horizontal bar) and interquartile range (box) with error bars representing most extreme values within 1.5 times the interquartile range.

Average daily movement among all fish was 2.3 ± 0.8 km with substantial daily variation ranging from undetectable movement (0 km) to over 15 km per day. The amount of daily movement varied significantly by season and transmitter type based on the optimal GLMM selected (Table S3). Seasonally, lake trout had significantly greater movement during fall (predicted daily movement = 1.20 km) compared to all other seasons (*p* < 0.001, Tukey) with approximately 45% further distances moved compared to spring (predicted daily movement = 0.85 km), 115% further compared to winter (predicted daily movement = 0.57 km) and 260% further compared to summer (predicted daily movement = 0.34 km). Spring and winter movements were comparable (*p* = 0.189, Tukey) and summer movements were statistically shorter than all other seasons (*p* < 0.001, Tukey; Figure 6). Fish tagged with V13 transmitters also moved significantly more (predicted daily movement = 0.85 km) than those with V9TP transmitters (predicted daily movement = 0.65 km, *p* = 0.001, GLMM; Figure 6); however, transmitter type was partially confounded by estimated length, considering fish less than 350 mm were only tagged using V9TP transmitters (Table 1). Origin was excluded from the optimal model but was included in three of the top five GLMMs (Table S3). The best‐fitting model that incorporated origin, which also included season and tag type, still indicated that origin had an insignificant difference between stocked (predicted daily movement = 0.72 km) and wild lake trout (predicted daily movement = 0.84 km, *p* = 0.257, GLMM; Figure S4).

### 3.3 Depth distribution

**FIGURE 7:**
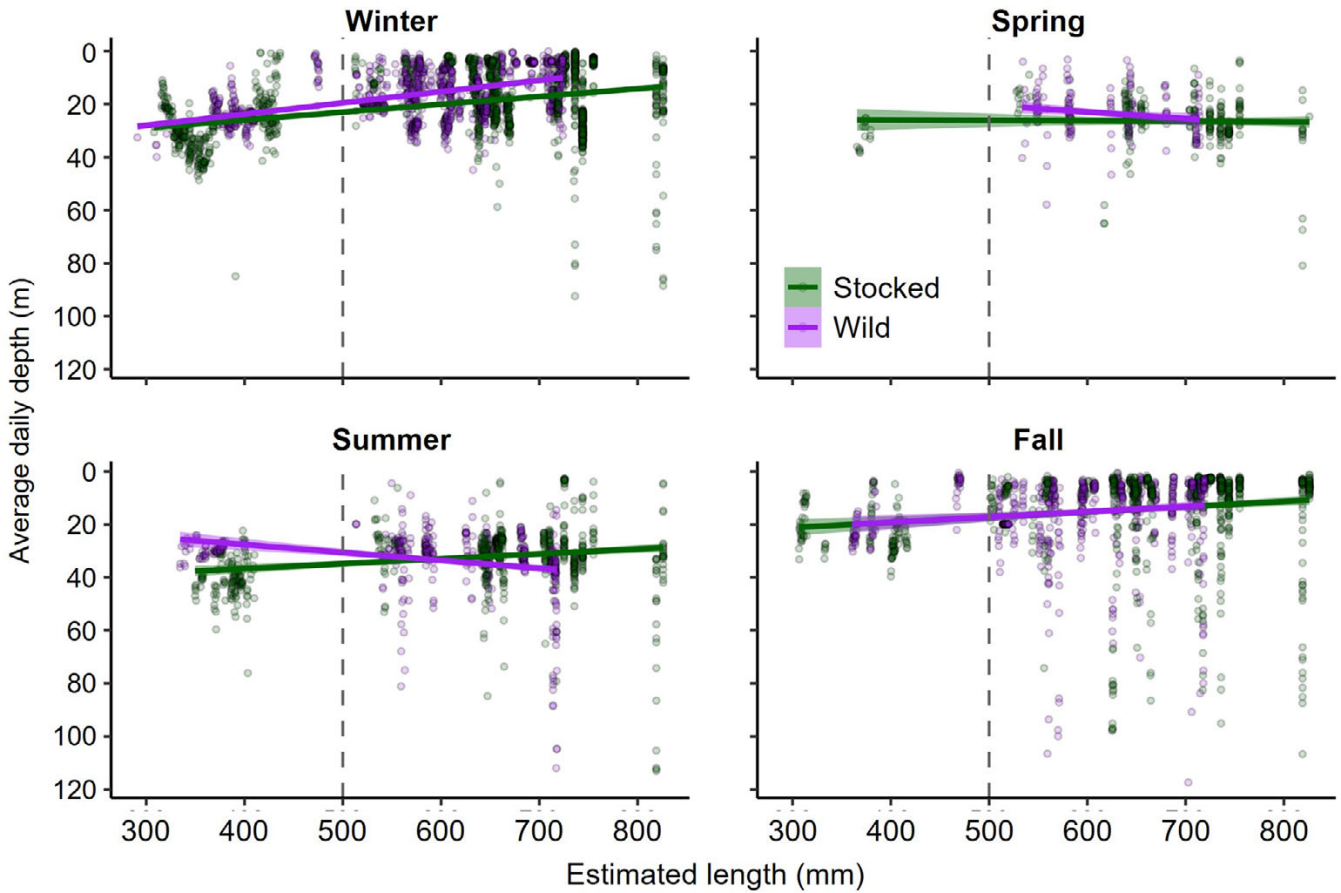
Average daily depth used by stocked and wild lake trout (*Salvelinus namaycush*) in response to estimated total length at the day of detection during each biological season. Depth data were filtered to randomly select 20 recordings for each fish daily. The linear line of best fit with 95% confidence intervals for stocked and wild fish is also included for each season. The grey vertical dashed line at 500 mm represents the approximate size at maturity for this lake trout population.

Lake trout depth use was highly variable, ranging between 0 and 120 m, with a maximum daily change in absolute depth of 109 m. The optimal GLMMs used to assess differences in average daily depth use included origin, season, the interactions between these variables and estimated length as significant covariates (Table S3). Overall, origin had a significant effect (*p* = 0.023, GAMM; Figure S4) with stocked lake trout occupying deeper habitats than wild fish (predicted depths of 20.5 and 19.9 m, respectively); however, all seasonal comparisons between stocked and wild fish were similar (*p* > 0.306, Tukey). Seasonal depths used by stocked and wild lake trout varied significantly, but differences among seasons were dependent on origin. For stocked lake trout, fish used significantly deeper habitats during summer (predicted average depth = 32.3 m) and spring (predicted depth = 26.4 m) compared to depths used during winter (predicted average depth = 17.8 m, *p* < 0.001, Tukey) and fall (predicted depth = 11.7 m, *p* < 0.001, Tukey). Depths used during winter were also significantly deeper than those used during fall (*p* < 0.001, Tukey) while depths used during summer and spring were similar (*p* = 0.978, Tukey; Figure 7). For wild lake trout, fish used significantly deeper habitats during summer (predicted depth = 33.6 m) compared to all other seasons (*p* < 0.013, Tukey). Depths used during spring (predicted average depth = 24.4 m) were also significantly deeper than depths used during fall (predicted average depth = 13.0 m, *p* < 0.001, Tukey) and winter (predicted average depth = 17.8 m, *p* < 0.001, Tukey), while depths used in these later two seasons were similar (*p* = 0.999, Tukey; Figure 7). Combined across seasons, smaller lake trout used deeper habitats than larger individuals (*p* < 0.001, GLMM; Figure 7). When grouped by maturity, immature lake trout occupied depths 55% deeper than mature fish (28.3 ± 13.0 m vs. 18.3 ± 11.8 m).

**FIGURE 8:**
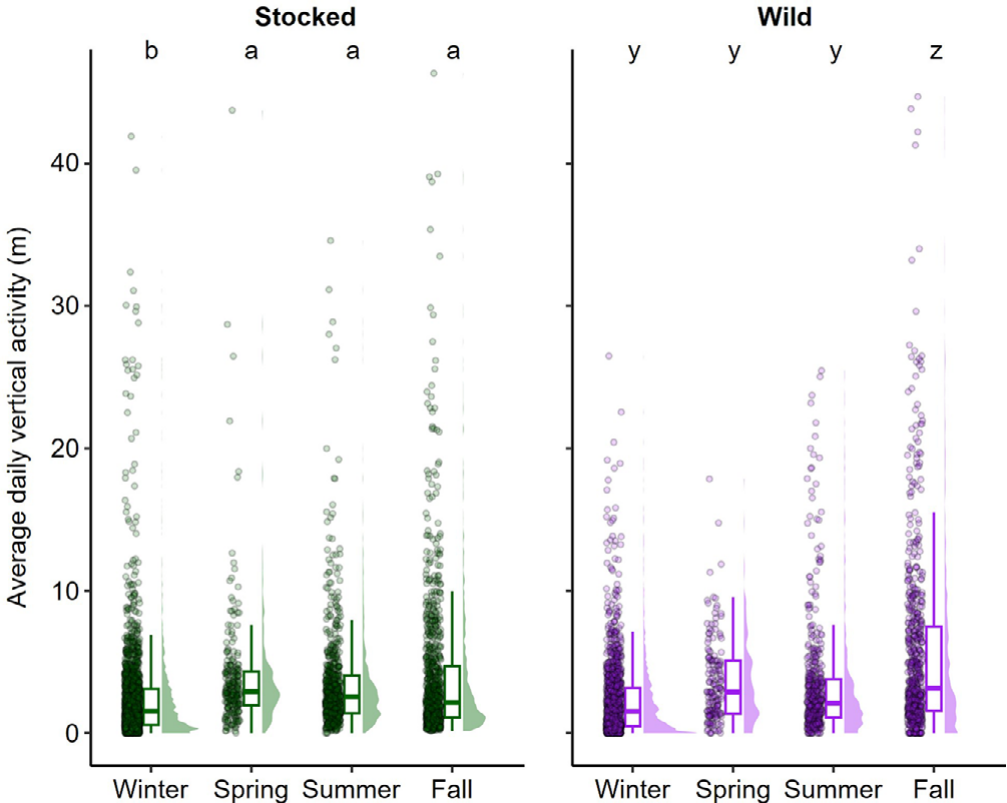
Average daily vertical activity measured as standard deviation of depth used by stocked and wild lake trout (*Salvelinus namaycush*) during each biological season detected. Depth data were filtered to randomly select 20 recordings for each fish daily. Significant differences among seasons by origin, based on estimated marginal means, are denoted by differing letters. Boxplots include the median values (horizontal bar) and interquartile range (box) with error bars representing most extreme values within 1.5 times the interquartile range.

Lake trout vertical activity was best explained by the GLMM that included origin, season and interactions between these two covariates (Table S3). Origin, however, had an insignificant effect overall (*p* = 0.914, GLMM; Figure S4). Pairwise comparisons by origin were also insignificant for each season (*p* > 0.139, Tukey), with the greatest difference during fall, when predicted daily vertical activities were 3.8 and 5.3 m for stocked and wild fish, respectively. Vertical activity varied significantly among seasons for stocked and wild lake trout, with differences dependent on origin. For stocked lake trout, significant differences were limited to pairwise comparisons involving winter such that daily vertical activity during that season (predicted activity = 2.4 m) was significantly less than activities during spring (predicted activity = 4.0 m, *p* = 0.010, Tukey), summer (predicted activity = 3.5, *p* = 0.004), and fall (predicted activity = 3.8 m, *p* < 0.001, Tukey; Figure 8). For wild lake trout, significant pairwise comparisons only included those that involved fall with significantly greater activity during that season (predicted activity = 5.3 m) compared to winter (predicted activity = 2.2 m, *p* < 0.001, Tukey), spring (predicted activity = 3.5 m, *p* < 0.001, Tukey) and summer (predicted activity = 3.2 m, *p* < 0.001, Tukey; Figure 8). All other pairwise comparisons among seasons for stocked and wild lake trout were insignificant (*p* > 0.103, Tukey; Figure 8).

## 4 DISCUSSION

Overall distributions, both horizontal and vertical, throughout Lake Champlain were largely consistent between stocked and wild lake trout at the seasonal scale. Both stocked and wild fish were widespread throughout the Main Lake regions and had similar occupancy and movement among these regions. The Northeast Arm and Malletts Bay also have deep habitats available but are known to experience extensive hypolimnetic hypoxia during summer (Smeltzer et al., 2012), which likely explains the rare use of these basins. Depth use, however, had small but notable differences between stocked and wild fish overall, with a tendency for stocked lake trout to use deeper habitats than wild fish. Previous research focusing on juvenile lake trout in Lake Champlain found the same pattern in which stocked juveniles were captured more frequently at deeper depths compared to wild fish (Wilkins & Marsden, 2021).

Movement patterns were also largely similar across sizes, suggesting immature (ages 3–5 years) and mature lake trout maintain similar spatial distributions except for depth occupancy, with immature fish typically occupying deeper habitats. The similar horizontal movements across lake trout sizes observed in this study are consistent with previous studies from large lakes that have demonstrated dispersal distances and home range sizes of both juvenile (ages 1–6 years) and adult lake trout are typically within 100 km (Binder et al., 2021). The negative relationship we observed between depth occupancy and lake trout size (i.e. larger lake trout occupy shallower habitats) is also consistent with previous descriptions of lake trout habitat use; immature lake trout typically remain close to the substrate whereas mature individuals are more likely to occupy pelagic and nearshore habitats (Marsden et al., 2021). Funnell et al. (2023), however, found larger lake trout were detected at deeper receivers in Lake Erie relative to smaller fish, but only during winter and spring, while there was no size effect during summer and fall. Their depth data, however, focused on adults (≥475 mm total length) and were limited to receiver depths as transmitters tracking lake trout did not include pressure sensors to determine actual depth occupied.

Our results demonstrated seasonal variability in lake trout distributions, although some of these differences were dependent on origin. Overall, both stocked and wild lake trout exhibited lower daily movement and increased dependence on deep habitats during summer and greater movement rates and dispersal during fall. These patterns have all been observed in previous studies and are considered to be associated with restricted habitat availability (i.e. cool and oxygenated water) during summer and migrations to spawning sites during fall (Binder et al., 2017; Blanchfield et al., 2023; Funnell et al., 2023; Ivanova et al., 2021). However, the significant interactions that we observed between season and origin suggest that stocked and wild lake trout have different seasonal shifts in their movement behaviours. Seasonal patterns in daily depth included more extreme differences between winter and fall for stocked lake trout compared to wild fish, while wild individuals had more extreme differences between spring and summer compared to stocked fish. The remaining two metrics with significant interactions between origin and season (maximum regional occupancy and daily vertical activity) both indicated that wild fish had greater movement throughout the water column and among lake regions during fall compared to other seasons, while stocked fish only experienced reduced vertical movement during winter compared to the other seasons. Additionally, seasonal variability in maximum regional occupancy was only observed for wild lake trout, which suggests that wild lake trout may have more dynamic horizontal movements throughout the year compared to stocked individuals. While nearly all the metrics we evaluated suggest no statistically significant differences based on origin, predicted differences similarly suggest consistent trends in which wild lake trout had greater movements compared to stocked individuals. Previous studies have also demonstrated more constrained dispersal of stocked salmonids compared to their wild counterparts in other systems (Chittenden et al., 2010; Kallio‐Nyberg et al., 2011). The ecological significance of seasonal differences in activity for wild lake trout or minor reductions in movement by stocked individuals is unclear. Understanding the extent of this effect may be important for interpreting seasonal population dynamics, such as increased relative abundance of wild fish in fall assessments compared to other seasons, which has been observed in Lake Michigan (Madenjian et al., 2023).

Changes in the extent of fish movements can have positive and negative consequences for individuals (e.g. energy conservation, predation risk, food availability) and whole populations (e.g. competition, mate selection, gene flow; Shaw, 2020). In Lake Champlain, significant increases in wild lake trout movements (greater vertical activity and less dependence on individual regions) during fall compared to other seasons may be important for reproductive success, considering lake trout make spawning migrations during this season. Lake trout in Lake Champlain tend to spawn in shallow waters (Ellrott & Marsden, 2004) but may move between shallow and deep habitats when transitioning among spawning locations. Therefore, the greater vertical activity for wild fish along with their increased movement among lake regions during fall compared to other seasons may suggest extensive movements among spawning sites. In contrast, the lack of significant changes in stocked lake trout movements during fall is consistent with recent findings that stocked adults use fewer spawning sites and stay at individual sites longer compared to wild fish (Futia, 2024). Additionally, wild lake trout have greater foraging efficiency and energy reserves compared to stocked individuals (Futia, Rinchard, & Marsden, n.d., in press), which may also be associated with different seasonal movement patterns between wild and stocked fish.

Behavioural differences between stocked and wild lake trout may indirectly result from population dynamics such as competitive interactions or directly from genetic differences or phenotypically plastic responses to ecological pressures associated with their corresponding rearing environments. Many studies have highlighted negative impacts of stocked fish on wild populations (McMillan et al., 2023; Naish et al., 2007), including the potential for stocked fish to alter movement and habitat selection of wild individuals through competitive displacement (Kostow, 2009). However, these interactions are typically observed in concentrated environments such as streams and rivers and have been documented when stocked fish are added to a system where wild fish are already present. In Lake Champlain, lake trout stocking was required to re‐establish a population and thus was a necessary precursor to restoring a wild population. Wild lake trout have recently appeared in large numbers where only resident stocked fish were present, and the large size of the lake and abundance of resources (i.e. food and habitat) suggests direct competition is unlikely. Thus, we speculate that the behavioural differences we observed are more likely the outcome of direct effects associated with different rearing environments. Hatchery rearing may cause relaxed natural selection that can lead to low reproductive success (e.g. number of spawning events and offspring survival) of the stocked population in natural environments (Araki et al., 2007; Thériault et al., 2011). In Lake Champlain for example, only a small proportion of the stocked lake trout (parental generation) contribute to the first generation (F1) produced in the wild, likely resulting in lower genetic diversity in the wild population (unpublished data). Alternatively, rapid behavioural adaptations to rearing environments can also result from substantial changes in gene expression, as demonstrated with hatchery‐reared fish (Christie et al., 2016; Nyman et al., 2020), which would not affect genetic diversity. Behavioural deficits including reduced swimming endurance and migration distance have been associated with hatchery rearing (Chittenden et al., 2010; McDonald et al., 1998; Pedersen et al., 2008) and could contribute to reduced movements that we observed with stocked individuals. Lastly, hatchery rearing can induce epigenetic modifications with potential for lasting effects on fish behaviour and biological functions (Le Luyer et al., 2017). Addressing these direct mechanisms individually is beyond the scope of this study but would provide valuable insight into the effects of behavioural maladaptations associated with hatchery rearing.

Some of the estimates for space use and extent of movement in this study may also have been influenced by factors including capture method, detection range variability, inconsistent and sparse receiver coverage, and autocorrelation among detections. The different capture methods used in this study may create a sampling bias in which passive methods (i.e. angling and gillnets) may have greater selection for individuals that tend to move more compared to active methods (i.e. bottom trawling) (Álvarez‐Quintero et al., 2021). Most fish were collected by angling, but many immature fish were collected using bottom trawls which confounds our comparisons based on maturity. This difference in capture methods by maturity may cause the tagged immature lake trout to disproportionately represent individuals that tend to move less compared to the adult population. While we did not account for capture method in our models due to limited sample sizes, we do not expect this bias to be strong considering lake trout are a mobile species (Binder et al., 2021). Seasonal differences in receiver detection efficiency may also contribute to these temporal patterns in lake trout distribution because thermal stratification can reduce detection range (Kuai et al., 2021; Wells et al., 2021). This pattern was seen in the Central Main Lake, where the detection range was greatest during fall and may result in increased probability of detection and consequently increase movement estimates. However, the trend was not consistent across lake regions and is unlikely to influence differences observed between stocked and wild fish considering any biases associated with the change in detection range would affect all fish equally. Inconsistent spatial receiver density and autocorrelation of detections can also create biases, particularly if fish occupy different areas, resulting in different probability of being detected (Brownscombe et al., 2022). Among our analyses, region use was likely the least affected considering the broad spatial and temporal evaluation of this metric. Proportional time spent in each region was also likely affected by low receiver coverage; however, the use of interpolated positions likely reduced error in estimated regional use (Futia et al., 2024). Average daily movements almost certainly underestimated actual movements due to our sparse receiver coverage and the inability to incorporate fine‐scale movements (Simpfendorfer et al., 2002). While this limitation would apply to all fish movements, individuals that remained in areas with lower receiver density would experience a greater bias in reduced movements, therefore differences in space occupied by stocked and wild lake trout may have led to a different extent of movement underestimation. For example, if stocked lake trout occupied areas with fewer receivers compared to wild fish, movement estimates for stocked fish would be underestimated to a greater extent than estimates for wild fish and may inaccurately contribute to the differences we observed. Differences in space use within regions in particular, which was not evaluated, could have an important effect while different use across regions was accounted for in our models by including region as a random effect. Depth distributions, which were evaluated daily, can also be influenced by limited receiver coverage as well as autocorrelation issues. These two issues together can lead to overrepresentation of vertical habitat where fish are observed by limiting observations to a constrained area. Our filtering and random subsampling of depth data would help alleviate this effect (Brownscombe et al., 2022), especially for fish with many (i.e. hundreds) daily detections whereas fish with fewer detections may still have inaccurate representations of daily depth distributions. The effects of these issues are also likely minimal considering most receivers were in proximity to deep habitat, with all but three stations detecting fish occupying depths deeper than 30 m. Thus, these issues should have minimal effects on the differences in observed depth distributions based on lake trout maturity or origin.

Although we observed behavioural differences between stocked and wild fish, the magnitude and effect of these differences appear to be minimal, especially considering stocked lake trout have successfully contributed to natural recruitment in Lake Champlain (Marsden et al., 2018). Spawning and free embryo production by stocked fish has been documented throughout the system since the early 2000s (Ellrott & Marsden, 2004; Marsden et al., 2005). It therefore seems unlikely that movement behaviours of stocked fish associated with hatchery rearing were a primary restriction on natural recruitment. In the Great Lakes, sea lamprey wounding, alewife (*Alosa pseudoharengus*) predation on free embryos and thiamine deficiency in free embryos caused by consumption of alewife by adult lake trout are considered to be major factors impeding natural recruitment (Fitzsimons et al., 2010; Muir et al., 2013). In Lake Champlain, survival of adult lake trout has been high despite high sea lamprey wounding (>35 wounds per 100 lake trout; Marsden et al., 2018; Hemmelgarn et al., 2022). Negative effects associated with alewife are also unlikely considering alewife were not observed in Lake Champlain until 2003 (Marsden & Langdon, 2012), therefore we construe that additional unknown factors likely contribute to the long period of recruitment failure. Nevertheless, our findings provide additional evidence that movement behaviours can depend on rearing environment and demonstrate long‐term effects of hatchery rearing on observed behaviours in stocked fish.

## AUTHOR CONTRIBUTIONS

M.H.F. conducted statistical analyses and drafted the paper. M.H.F. and J.E.M. developed the ideas and study design, acquired funding and data, and contributed to the final version of the manuscript.

## FUNDING INFORMATION

Funding for this project was primarily provided by Lake Champlain Sea Grant (Grant ID # 000032817) and the Great Lakes Fishery Commission with funds made available to Lake Champlain by Senator Patrick Leahy (Grant ID # 2018_MAR_95003 & 2023_MAR_95003). Additional funding was provided by the Great Lakes Fishery Commission (Grand ID # 2013_BIN_44024) by way of Great Lakes Restoration Initiative appropriations (Grant ID # GL‐00E23010).

## Supporting information


**DATA S1.** Supporting Information.

## Data Availability

The raw data used for analyses in this manuscript will be made available by the corresponding author, without undue reservation, upon reasonable request.
