# Supplementary material for: Movement behaviours of stocked and wild lake trout Salvelinus namaycush determined using acoustic telemetry
Source: J Fish Biol. 2025 May 2;107(2):613–27. doi: 10.1111/jfb.70071 (PMC12360149; doi:10.1111/jfb.70071)
Supplement: Supplementary file 1 — DATA S1. Supporting Information. [file JFB-107-613-s001.docx]

**Supporting Information**

Supplementary Text

*Range Testing*

Range tests were conducted intermittently in the Northeast Arm, Malletts Bay, and the three Main Lake regions during 2021 and 2022. Tests involved deploying transmitters at known distances from a target receiver for approximately 30 minutes, with transmitters suspended 3-5m above the lake bottom. In the Central Main Lake there were two receivers within the range of the test transmitters that provided replicated data. Range tests included V9 and V13 transmitters programmed to transmit at intervals of either seven (V9 only) or 15 (V9 and V13) seconds. Transmitters set to a seven-second interval had low power transmission strength (146 dB re 1 µPa at 1 m) and those set to a 15-second interval were programed to high power (151 and 152 dB re 1 µPa at 1 m). Range test data were used to calculate the distance from receivers associated with 50% detection efficiency (DE; Brownscombe et al., 2020); however, the estimated DEs were largely dependent on detections from high power transmitters and thus were inappropriate for informing the detection range of the transmitters used to track the lake trout in this study, which were all programmed for low power to extend transmitter battery life. Therefore, range test results were only used to identify broad trends in detection range among transmitter types, regions, and seasons that informed our interpretation of lake trout movement patterns.

Range test transmitter detection distances associated with 50% DE differed based on the transmitter size (V9 versus V13), lake region, and season. Overall, V13 transmitters could be detected by receivers at further distances than V9 transmitters, with 1-24% increases in 50% DE distances. Comparisons between range test transmitters with different power settings (i.e., low versus high power) were not possible due to confounding seasonal differences in deployments. The greatest DE distances among regions were observed in the Central Main Lake; however, there were consistent differences between the two receivers used simultaneously for range tests in the region with 50% DE distances differing by 27-54%. The distance associated with 50% DE in Malletts Bay was further than that of the South Main Lake during winter (30-51% increase depending on tag type); however, the opposite trend occurred in spring with better DE in the South Main Lake compared to Malletts Bay (25% increase). There were no consistent seasonal trends in DE among regions, although both receivers in the Central Main Lake experienced better DE during fall compared to spring for both the V9 (23-43% increase) and V13 (30-58% increase) transmitters (Supplementary Table 2).

Supplementary Table 1. Complete lake trout (*Salvelinus namaycush*) collection data with sample size (N) by origin, life stage, collection method, transmitter type, capture region (Main Lake North = MLN, Main Lake Central = MLC, Main Lake South = MLS), and months collected.

| Origin | Life stage | Method | Transmitter | Region | Month | N |
| --- | --- | --- | --- | --- | --- | --- |
| Stocked | Juvenile | Trawl | V9TP | NML | Aug | 2 |
|  |  |  |  |  | Oct | 4 |
|  |  |  |  | CML | Oct | 3 |
|  |  |  |  | SML | Oct | 2 |
|  | Sub-adult | Angling | V9TP | CML | Aug | 1 |
|  |  |  |  |  | Sep | 1 |
|  |  |  | V13 | CML | Sep | 4 |
|  |  |  |  |  | Oct | 2 |
|  |  |  |  |  | Nov | 2 |
|  | Adult | Angling | V9TP | CML | Jul | 2 |
|  |  |  |  |  | Sep | 13 |
|  |  |  | V13 | CML | Aug | 1 |
|  |  |  |  |  | Sep | 5 |
|  |  |  |  |  | Oct | 1 |
|  |  |  |  |  | Nov | 9 |
|  |  | Gill net | V13 | CML | Oct | 4 |
|  |  |  |  |  |  |  |
| Wild | Juvenile | Trawl | V9TP | CML | Oct | 5 |
|  | Sub-adult | Angling | V9TP | CML | Jul | 5 |
|  |  |  | V13 | CML | Sep | 5 |
|  |  |  |  |  | Oct | 8 |
|  |  |  |  |  | Nov | 1 |
|  |  | Gill net | V13 | CML | Oct | 1 |
|  | Adult | Angling | V9TP | CML | Sep | 5 |
|  |  |  | V13 | CML | Nov | 1 |
|  |  | Trawl | V9TP | CML | Aug | 2 |
|  |  |  | V13 | CML | Nov | 1 |

Supplementary Table 2. Comparison of distances (m) associated with 50% detection efficiency (DE) among lake regions, seasons, and Innovasea transmitters (V9 and V13). Lake regions are abbreviated as follows: North Main North = NML, Central Main Lake = CML, South Main Lake = SML, MAB = Malletts Bay, and NEA = Northeast Arm. Each row represents a different receiver station for a given region/season combination.

| Region | Season | V9 DE | V13 DE |
| --- | --- | --- | --- |
| NML | Spring | 881 | 996 |
|  | Summer | 699 | 710 |
|  | Fall | 741 | 777 |
| CML | Spring | 914 | 919 |
|  |  | 716 | 724 |
|  | Fall | 1,307 | 1,449 |
|  |  | 882 | 942 |
| SML | Winter | 886 | 954 |
|  | Spring^†^ | 883 |  |
| NEA | Summer | 1,084 | 1,227 |
| MAB | Winter | 1,156 | 1,436 |
|  | Spring^†^ | 704 |  |

^†^Only V9TP transmitters programmed to low transmission strength were used for range tests.

Supplementary Table 3. Model selection table for the top five generalized linear mixed models for evaluating significant covariates of lake trout horizontal (region use, maximum regional occupancy, and daily movement) and vertical movement (average depth and vertical activity). Parameter estimates are included for continuous variables that were included in corresponding models and a plus sign (+) indicates categorical parameters that were included. Estimated size was evaluated as the estimated total length (mm) of fish at the time of detection for all metrics except maximum regional occupancy, which used the estimated maturity status (immature or mature) instead. Tag type was also not included in either metric for vertical activity as only one tag type (V9TP) was included. Models are ordered by change in Akaike information criterion compared to the optimal model (ΔAIC) and the degrees of freedom based on number of covariates included in each model is also included (df). The optimal model selected based on ΔAIC and model parsimony (lowest df) are in bold text.

| Metric | Intercept | Origin | Season | Origin*Season | Estimated size | Tag type | df | ΔAIC |
| --- | --- | --- | --- | --- | --- | --- | --- | --- |
| Region  use | 0.71 | + | - | - | - | - | 5 | 0.00 |
|  | 0.44 | + | - | - | 4.36E-04 | - | 6 | 0.09 |
|  | **0.75** | **-** | **-** | **-** | **-** | **-** | **4** | **0.74** |
|  | 0.60 | - | - | - | 2.63E-04 | - | 5 | 2.02 |
|  | 0.71 | + | - | - | - | + | 6 | 2.04 |
|  |  |  |  |  |  |  |  |  |
| Maximum regional occupancy | 2.00 | + | + | + | + | - | 13 | 0.00 |
|  | **1.76** | **+** | **+** | **+** | **-** | **-** | **12** | **0.92** |
|  | 1.94 | + | + | + | + | + | 14 | 1.62 |
|  | 1.70 | + | + | + | - | + | 13 | 2.29 |
|  | 1.75 | + | - | - | - | - | 6 | 2.34 |
|  |  |  |  |  |  |  |  |  |
| Daily movement | **0.29** | **-** | **+** | **-** | **-** | **+** | **10** | **0.00** |
|  | 0.26 | + | + | - | - | + | 11 | 0.76 |
|  | 0.21 | + | + | + | - | + | 14 | 0.79 |
|  | 0.21 | - | + | - | 1.38E-04 | + | 11 | 1.85 |
|  | 0.08 | + | + | - | 2.91E-04 | + | 12 | 2.04 |
|  |  |  |  |  |  |  |  |  |
| Average depth | **3.95** | **+** | **+** | **+** | **-2.01E-03** | **NA** | **14** | **0.00** |
|  | 3.92 | - | + | - | -2.14E-03 | NA | 10 | 35.47 |
|  | 3.96 | + | + | - | -2.15E-03 | NA | 11 | 36.38 |
|  | 2.77 | + | + | + | - | NA | 13 | 44.92 |
|  | 2.69 | - | + | - | - | NA | 9 | 85.67 |
|  |  |  |  |  |  |  |  |  |
| Vertical activity | **0.99** | **+** | **+** | **+** | **-** | **NA** | **14** | **0.00** |
|  | 1.20 | + | + | + | -3.62E-04 | NA | 15 | 1.27 |
|  | 0.98 | - | + | - | - | NA | 10 | 29.87 |
|  | 1.25 | - | + | - | -4.49E-04 | NA | 11 | 30.74 |
|  | 0.94 | + | + | - | - | NA | 11 | 31.04 |


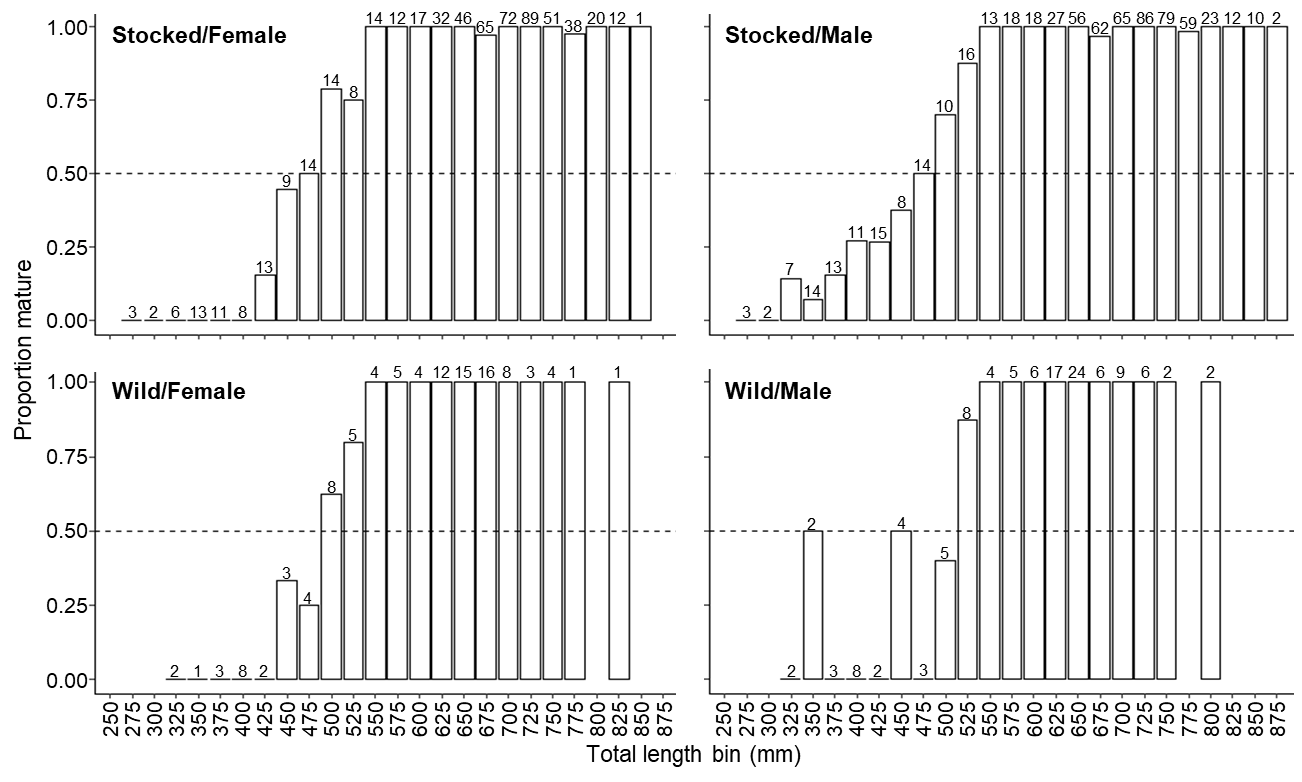
Supplementary Figure 1. Proportion of lake trout (*Salvelinus namaycush*) at maturity grouped by origin and sex and binned into 25 mm groups. Sample size for each bin is included above the corresponding box. Sex could not be determined for immature fish and therefore these individuals are added to both female and male distributions. The dotted line at 0.50 represents 50% maturity at a given length.


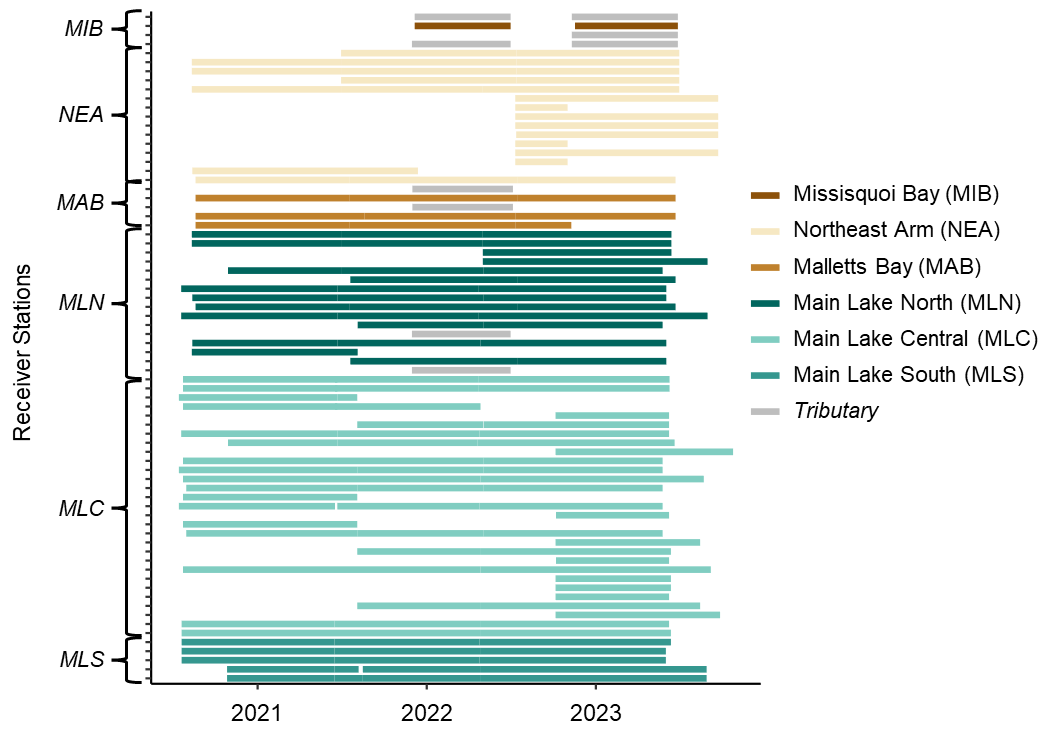


Supplementary Figure 2. Deployment periods for individual receiver stations between summer 2020 and summer 2023. Receiver stations are arranged by latitude within lake regions. Gray bars represent receiver stations located in tributaries that flow into the corresponding region.


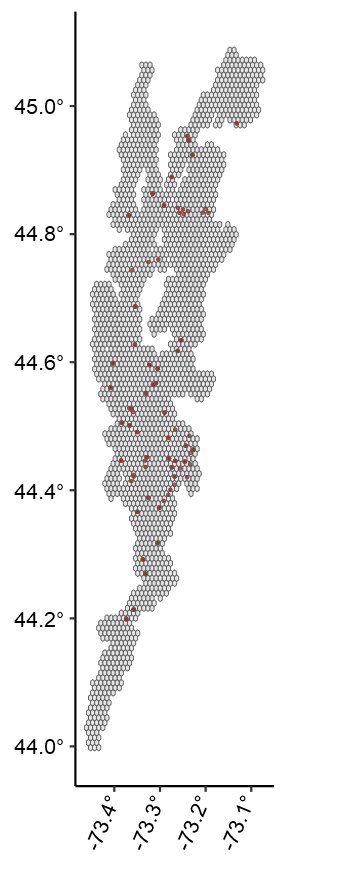


Supplementary Figure 3. Hexagonal grid with 1 km polygons overlaying Lake Champlain, excluding the South Lake, used for aggregating 1-hr centers of activity. Acoustic receiver locations with a 500 m radius, corresponding to approximately 30% detection range (Pinheiro et al. 2017), are included as red points.


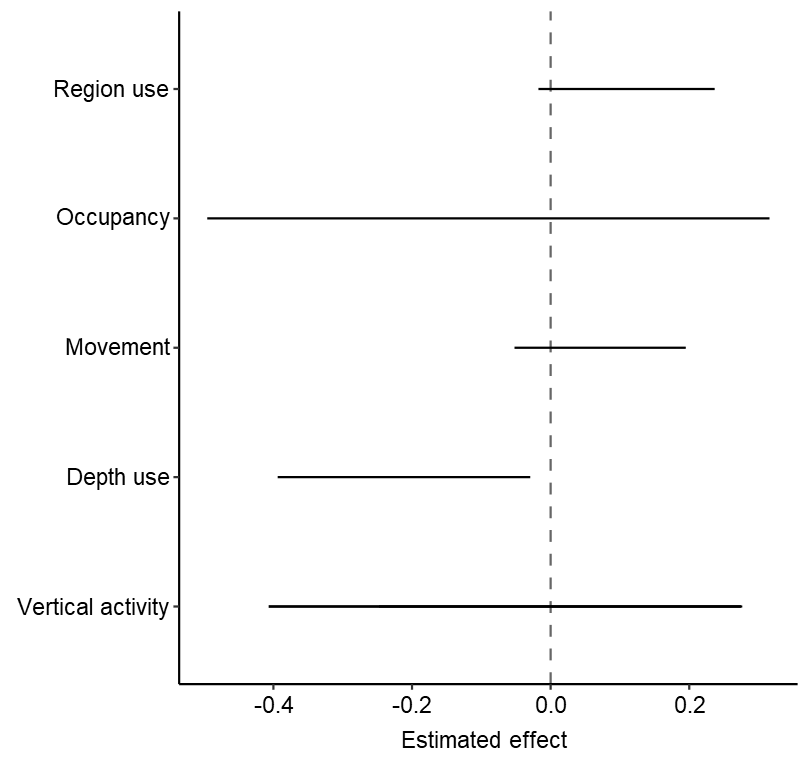
Supplementary Figure 4. Ninety-five percent confidence intervals for comparisons between stocked and wild lake trout for each of the five analyses in our study. Analyses included number of regions used within a season (region use), maximum regional occupancy within a season (occupancy), average daily movement (movement), average daily depth (depth use), and vertical activity. Intervals are based on estimates from the generalized linear model with the lowest Akaike information criterion score that included origin. Negative values indicate greater estimates for stocked fish whereas positive values indicate greater estimates for wild fish. Significant differences were observed when confidence intervals did not cross zero, designated by the vertical dashed line.
